# Supplementary figures and images for: The Proteomic Landscape of CTNNB1 Mutated Low-Grade Early-Stage Endometrial Carcinomas
Source: Cells. 2025 Oct 27;14(21):1676. doi: 10.3390/cells14211676 (PMC12610012; doi:10.3390/cells14211676)

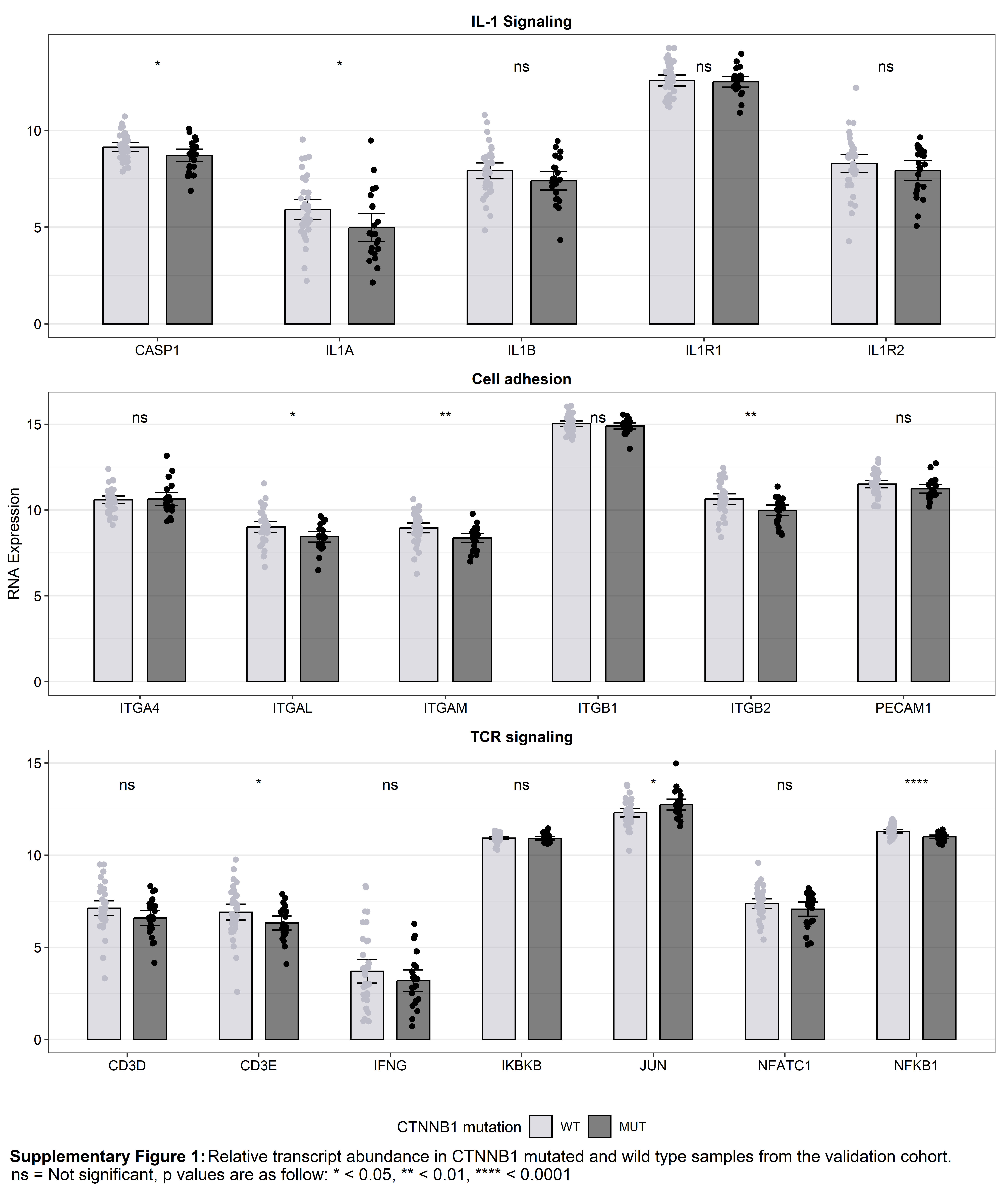

Supplement: Supplementary file 1 [file cells-14-01676-s001.zip › Supplementary_Figure_S1_rev.png]
